# Hospital Employees and Unemployment Insurance

**Published:** 1921-02-12

**Authors:** 


### Hospital Employees and Unemployment Insurance.

Decisions under Section 10 of the Unemployment Insurance Act have been conveyed to the Hon. Secretary of the British Hospitals Association in a letter from Mr. W. R. L. Blakiston, of the Ministry of Labour's Employment Department. They concern specified employments on the male and female staffs of the Metropolitan Asylums Board. In three cases—that of a person employed as a laboratory attendant to prepare nutrient broth and other culture media for bacteriological purposes, employment as a messenger, and employment as a fireman to attend to the fire-engines and fire apparatus at the Board's institutions—the Minister has decided that the employment is employment within the meaning of the Act. In fifteen cases, which include purely domestic work such as that of cook, kitchenmaid, daily woman, etc., at one of the Board's institutions, a foreman or other porter who assists to carry patients, a sempstress to mend underwear, etc., belonging to the staff and patients, a coachman who also does certain indoor work, the decision is that the employment is not employment within the meaning of the Act. The same decision is given in the case of employment as a gardener for the garden attached to a private residence. On the information before the Minister it would appear that the employment of the resident indoor porters, ward maids, and laundry maids in voluntary hospitals is covered in principle by certain of these decisions, and in this event no contributions are payable, and that similarly employment in voluntary hospitals of persons upon duties analogous to those described in the remaining decisions would be covered in principle by those decisions. If any contributions have been paid pending these decisions in respect of any persons the Minister will be prepared to consider an application under Section 28 for a refund. A further point to be noted is that the Minister is advised that hospitals supported out of voluntary contributions are not local or other public authorities within the meaning of paragraph (*d*) of Part II. of the First Schedule to the Act (Excepted Employments). We advise all hospital secretaries to procure a copy of the Minister's circular, which they can do by writing to the Hon. Secretary of the British Hospitals Association, Stapley House, 33 Bloomsbury Square, W.C. 1.

The Minister of Labour has further intimated that no general ruling can be given as to the possibility of any class of nurses being excepted under paragraph (*h*) of Part II. of the First Schedule (Excepted Employments, otherwise than by way of manual labour and at a rate of remuneration exceeding in value £250). The rate of remuneration must be a question of fact in each particular case, and the position in this respect is the same under the Unemployment Insurance Act as it is under the National Health Insurance Acts. The decision in regard to the sister, trained nurse, and probationer has not yet been given.

